# Exploring the relationship between α-actinin-3 deficiency and obesity in mice and humans

**DOI:** 10.1038/ijo.2017.72

**Published:** 2017-04-11

**Authors:** P J Houweling, Y D Berman, N Turner, K G R Quinlan, J T Seto, N Yang, M Lek, D G Macarthur, G Cooney, K N North

**Affiliations:** 1 Institute for Neuroscience and Muscle Research (INMR), The Children’s Hospital at Westmead, NSW, Australia; 2 Department of Pediatrics, The University of Melbourne, Victoria, Australia; 3 Murdoch Childrens Research Institute, Parkville, Victoria, Australia; 4 Discipline of Child and Adolescent Health, Sydney Medical School, The University of Sydney, New South Wales, Australia; 5 Department of Clinical Genetics, Royal North Shore Hospital, New South Wales, Australia; 6 Faculty of Science, School of Biotechnology and Biomolecular Sciences, UNSW Australia, Sydney, New South Wales, Australia; 7 Diabetes and Metabolism Program, Garvan Institute of Medical Research, Sydney, New South Wales, Australia; 8 Analytic and Translational Genetics Unit, Massachusetts General Hospital, Boston, MA, USA; 9 Program in Medical and Population Genetics, Broad Institute of MIT and Harvard, Cambridge, MA, USA; 10 Charles Perkins Centre, Sydney Medical School, The University of Sydney, New South Wales, Australia

## Abstract

Obesity is a worldwide health crisis, and the identification of genetic modifiers of weight gain is crucial in understanding this complex disorder. A common null polymorphism in the fast fiber-specific gene *ACTN3* (R577X) is known to influence skeletal muscle function and metabolism. α-Actinin-3 deficiency occurs in an estimated 1.5 billion people worldwide, and results in reduced muscle strength and a shift towards a more efficient oxidative metabolism. The X-allele has undergone strong positive selection during recent human evolution, and in this study, we sought to determine whether *ACTN3* genotype influences weight gain and obesity in mice and humans. An *Actn3* KO mouse has been generated on two genetic backgrounds (129X1/SvJ and C57BL/6J) and fed a high-fat diet (HFD, 45% calories from fat). Anthropomorphic features (including body weight) were examined and show that *Actn3* KO 129X1/SvJ mice gained less weight compared to WT. In addition, six independent human cohorts were genotyped for *ACTN3* R577X (Rs1815739) and body mass index (BMI), waist-to-hip ratio-adjusted BMI (WHRadjBMI) and obesity-related traits were assessed. In humans, *ACTN3* genotype alone does not contribute to alterations in BMI or obesity.

## Introduction

The sarcomeric α-actinin’s (actinin-2 and -3) are major components of the skeletal muscle Z-line and were thought to provide structural support during muscle contraction. We now know that they are also key adaptor proteins interacting with many structural, signaling and metabolic proteins.^1^ In both humans and mice, α-actinin-2 is ubiquitously expressed in all muscle fibers, whereas α-actinin-3 (encoded by the *ACTN3* gene) has evolved to be predominantly expressed in fast (type 2) glycolytic muscle fibers. We identified a common null polymorphism in the *ACTN3* gene (R577X); homozygosity for the X-allele (577XX) results in the complete absence of α-actinin-3 in 16–18% of the global population.^2, 3^ The absence of α-actinin-3 (577XX) significantly influences skeletal muscle function in both elite athletes and the general population;^4, 5^ specifically, α-actinin-3 deficiency is detrimental to sprint performance, and is associated with reduced muscle strength and mass.

To further understand the role of α-actinin-3 in skeletal muscle, an *Actn3* knockout (KO) mouse has been developed that replicates many of the phenotypes reported in humans.^6, 7, 8^ Compared to WT, *Actn3* KO mice show lower total body weight (due to a reduction in muscle mass as a result of smaller type 2B fiber size), along with reduced grip strength and increased endurance capacity.^6, 7^ Dual-energy X-ray absorptiometry (DXA) in young *Actn3* KO mice also shows a reduction in bone mineral density (BMD), and fat-free mass, while total fat mass in the KO is maintained.^9^ α-Actinin-3 deficiency results in altered skeletal muscle metabolism, with decreased glycogen phosphorylase activity and a shift in the metabolic profile of fast glycolytic fibers towards a ‘slower’ oxidative phenotype without a change in fiber type.^1^

Human association studies exploring the effect of *ACTN3* genotype on body mass and obesity have provided mixed results. One study assessed body composition by DXA in 848 adults (*n*=454 men and 394 women, aged 22–90 years) found that α-actinin-3 deficiency was associated with reduced fat-free muscle mass, total body weight, BMI and total body fat mass in women.^10^ A second study of Caucasian men (*n*=100, aged 60–70 years) found no significant difference in body mass or fat-free mass with *ACTN3* genotype, but results for fat mass were not discussed.^11^ A third study in adolescent boys, which used skin fold measurement as an estimation of body fat, also found no correlation between *ACTN3* genotype and fat mass.^4^ Most recently, an investigation into the effect of *ACTN3* genotype in overweight males and females (*n*=177, 58–63 years, BMI >29 kg m^−2^) highlighted a higher proportion of α-actinin-3-deficient type 2 diabetic individuals compared to normal glucose tolerance (NGT) controls (577XX 24 T2D vs 12% NGT).^12^ However, a lack of congruent studies, small cohort sizes, wide age ranges and differences in methods used to assess body composition and adiposity make it difficult to determine a clear effect of *ACTN3* genotype in these studies.

## Results and discussion

Skeletal muscle is a fundamental metabolic ‘organ’ making up the largest individual body mass fraction in non-obese adults, and accounting for ~40% of total body mass in men and 33% in women.^13^ Genetic variants, which influence skeletal muscle metabolism and energy expenditure, such as the *ACTN3* R577X, may also affect ‘whole body’ metabolic phenotypes such as obesity. Genome-wide association studies (GWAS) have identified up to 77 obesity-susceptibility loci,^14^ although supporting evidence (in the form of replication studies and functional validation) is available for very few. For this reason, the use of hypothesis-driven candidate analyses to assess the functional impacts of known genetic variants remains a vital tool in the search for genetic factors that contribute to obesity.

Since α-actinin-3 deficiency is common in the general population (~1.5 billion individuals worldwide) and its absence results in a significant shift in muscle metabolism, we sought to determine whether *ACTN3* genotype influences weight gain and the risk of obesity in large well-characterized human cohorts. In addition, using the two different *Actn3* KO mouse strains, we further examined the effect of a HFD (45% calories from fat) to examine the impact of *Actn3* genotype on weight gain, while controlling for variations in environment, diet and genetic background/ethnicity.

**Figure 1:**
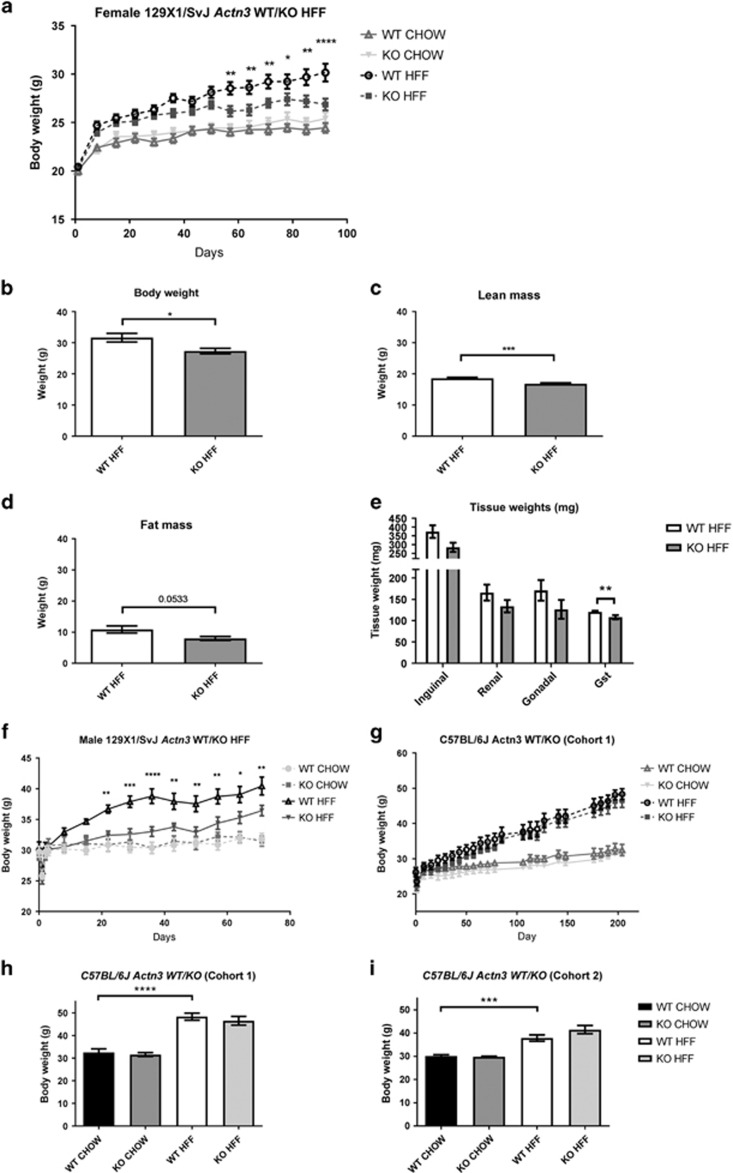
Female 129X1/SvJ *Actn3* KO mice show reduced body weight following 92 days of high-fat feeding (**a**, **b**). DXA analysis of HFF *Actn3* KO mice show reduced lean and fat mass compared to HFF WT mice (**c**, **d**). Individual tissue weights for inguinal, renal, and gonadal fat pads as well as the gastrocnemius (GST) muscle show that HFF *Actn3* KO mice have reduced fat and muscle mass (**e**). Male 129X1/SvJ *Actn3* KO mice also show reduced weight gain following 71 days on a high-fat diet (**f**). However, two independent HFF male C57BL/6J *Actn3* WT and KO cohorts show significant weight gain compared to CHOW-fed controls (**f**–**i**), but no difference between genotype following HFF. For the Female 129X1/SvJ cohort: WT HFF *n*=22; KO HFF *n*=18; WT CHOW *n*=6; KO CHOW *n*=9. Male 129X1/SvJ mice: WT HFF=7; KO HFF=5; WT CHOW=3; KO CHOW=4. Male C57BL/6J (cohort 1) were fed a HFD for 204 days and consisted of: WT HFF *n*=6; KO HFF *n*=6; WT CHOW *n*=4; KO CHOW *n*=5; Cohort 2 were fed a HFD for 94 days and consisted of WT HFF *n*=11; KO HFF *n*=12; WT CHOW *n*=10; KO CHOW *n*=10. All the data are mean values±s.e.m. and statistical analyses were performed using two-way ANOVA in GraphPad prism (version 7, La Jolla, CA, USA). Significance is represented by *P<0.05, **P<0.01, ***P<0.001, ****P<0.0001.

Following high fat-feeding (HFF), both 129X1/SvJ and C57BL/6J mouse strains show a significant increase in body weight, compared to CHOW-fed control mice (Figure 1a, f–i). Weight gain was significantly reduced in female 129X1/SvJ *Actn3* KO (Figures 1a–e). This difference in body weight was due to a significantly lower lean mass (Figure 1c) and a trend towards lower fat mass (Figure 1d, *P*=0.0533). Isolated fat pads and skeletal muscle (gastrocnemius) weights highlight the reduction in lean mass and fat mass in the female 129X1/SvJ *Actn3* KO HFF mice compared to WT HFF (Figure 1e). In addition, *Actn3* KO HFF 129X1/SvJ male mice also show reduced body weight compared to WT HFF mice (Figure 1f). This suggests that α-actinin-3 deficiency is protective against weight gain on a HFD in the 129X1/SvJ mouse strain. We repeated this analysis in two separate C57BL/6J *Actn3* cohorts fed with a HFD, generated by either specialty feeds (Cohort 1; Figures 1g and h) or the Garvan Institute (Cohort 2, Figure 1i). Significant increases in body weight compared to CHOW-fed controls were demonstrated in both the cohorts (Figures 1h and i). However, no differences in weight gain were observed when comparing WT and *Actn3* KO mice in either C57BL/6J cohort.

Technically, the *ACTN3* R577X loss-of-function polymorphism is excluded from GWAS analyses as it is annotated in the *Ensembl* reference gene set as a polymorphic ‘pseudogene’.^15^ Previous studies have shown mixed results relating to an effect of *ACTN3* genotype on traits like BMI and body weight. We therefore sought to assess a group of well-annotated human cohorts and the *Actn3* KO mouse to further explore the potential association between *ACTN3* R577X and obesity/weight gain.

We analyzed two case control populations (Ottawa and WTCCC) and three genome-wide association studies (DIAGRAM, GIANT and GIANTextreme) for an effect of *ACTN3* R577X polymorphism. Of the case:control cohorts (Ottawa and WTCCC), there was a lower frequency of obese α-actinin-3-deficient males compared to lean controls (577XX 11 obese vs 25% lean, *P*=0.02) in the Ottawa cohort. However, the larger WTCCC cohort did not show a significant association between *ACTN3* 577X allele and BMI (*P*=0.095). In addition, the most recent GWAS from the DIAGRAM consortium showed no association between the 577X allele, BMI or obesity (*P*=*0.18)*. Similarly, the GIANT consortium cohorts (GIANT and GIANTextreme) failed to highlight a difference in *ACTN3* 577X allele frequency and BMI, waist-to-hip ratio-adjusted BMI (WHRadjBMI) or Obesity class traits (*P*=0.6996; *P*=0.180; *P*=0.075; *P*=0.392, respectively; Table 1).

These data suggest that *ACTN3* genotype (in both humans and mice) does not strongly influence BMI, weight gain or obesity related traits. However, it is important to consider that the absence of α-actinin-3 (577XX) is a variation in 'normal’ muscle function. In elite athletes and the general population, the *ACTN3* 577X allele is thought to account for between ~1–2.3% of the variance in sprint time, respectively.^4, 16^ With the exception of *FTO*, very few polymorphisms have been consistently linked to the development of complex disorders like weight gain, obesity and T2D. Similarly, it is thought that obesity is the result of a combination of yet to be characterized genes that may influence this phenotype.

In this study, we found that 129X1/SvJ *Actn3* KO mice gained significantly less weight compared to WT on a HFD, despite similar caloric consumption and activity levels (data not shown). However, a similar genotype difference was not observed in the C57BL/6J *Actn3* KO mice. C57BL/6J is a commonly used strain in obesity studies due to their inherent susceptibility to develop obesity, type 2 diabetes and atherosclerosis on a HFD, suggesting that this strain may already carry a combination of genetic variants that diminished any contributing effects *ACTN3* may play in obesity. We have previously demonstrated a wide range of other phenotypes attributable to α-actinin-3 deficiency (including reduced grip strength and improved endurance capacity) that robustly reproduced on both the 129X1/SvJ and C57BL/6J genetic backgrounds.^8^ In mouse models as in humans, there is considerable variability in the onset and progression of obesity in different mouse strains.^17^ While we have controlled for environment and diet in the current study, we propose that differences in genetic background may explain the lack of replication between the two strains.

Differences in genetic background (ethnicity/population diversity) are thought to be a major contributor to identifying and validating the effects of candidate genes in human association studies of obesity. This current analysis focus on European populations. Studies utilizing additional populations (such as East Asians, Hispanic and Pacific Islander nations) may provide additional information currently not captured by this study. It is difficult to control for differences in environment (diet and exercise) as well as variables associated with the techniques used to measure body composition. Large population-based studies attempt to capture this information; however, the commonly used BMI measurement may not be sensitive enough to detect more subtle changes in adiposity. More sensitive physical measures of fat and body mass, such as DXA, may also provide additional insight into the role of α-actinin-3 in body composition in humans.

This current study provides mixed support for a role of *ACTN3* R577X in weight gain/obesity in individuals of European descent. However, this does not exclude a potential role in obesity-related diseases such as chronic heart failure (CHF) and T2D. Recently, the absence of α-actinin-3 has been linked to poor survival in patients with CHF.^18^ Similarly, an increase in the frequency of α-actinin-3-deficient individuals has been observed in a small cohort of T2D patients, irrespective of BMI.^12^ Tantalizing differences in weight gain (129X1/SvJ *Actn3* KO) and select human cohorts (Ottawa) suggest further analysis will be required before excluding *ACTN3* genotype as a possible genetic modifier of obesity and weight gain.

## Figures and Tables

**Table 1:** Human cohort analyses

*Cohort*	*Participants*	*No. of individuals*	*Trait/effect of ACTN3*	*Statistics*	*Cohort citation*
*Case: Control populations*					
** **Ottawa	Age: 33–60		Obesity class III (BMI>39):	Male *P*=0.02	^19^
	Obese: BMI=49	*N*=372 obese (36% Male)	Reduced frequency in obese XX males (*n*=15) compared to lean controls (*n*=31). No genotype differences between obese XX females (*n*=45) and lean controls (*n*=37).	Female *P*=0.5	
	Lean: BMI=19.4	*N*=372 lean (37% Male)			
** **WTCCC	Age: 48–69		BMI/no difference between genotypes	*P*=0.095	^20^
	Obese: BMI >30	*N*=1924 obese	Coefficient of association −0.64,		
	Lean: BMI <25	*N*=2938 controls	(95% CI −1.4 to −0.112).		
					
*Genome-wide association study (GWAS) populations*					
** **Diagram	Male	*N*=101193	BMI/No difference between genotypes	*P*=0.18	^21^
** **GIANT (public dataset)	Male	*N*=72495	BMI/No difference between genotypes	*P*=0.696	^22^
** **GIANT	Male	*N*=39053	WHRadjBMI/no difference between genotypes	*P*=0.180	^23^
** **GIANT (extremes)	Male	*N*=3062 obese; N=3752 controls	WHRadjBMI/no difference between genotypes	*P*=0.075	^24^
		*N*=1708 obese; *N*=34395 controls	Obesity Class III (BMI>39)/no difference between genotypes	*P*=0.392	

Abbreviations: BMI, Body mass index; N, number of participants; WHRadjBMI, Waist-to-hip ratio-adjusted BMI.
